# Utilizing the Leap Motion Controller for skill tracking in surgical training: solving line-of-sight issues

**DOI:** 10.1016/j.sopen.2025.06.009

**Published:** 2025-06-25

**Authors:** V.E.E. Kleinrensink, R.H.M. Goossens, J.F. Lange, L.W. Kranenburg, G.J. Kleinrensink

**Affiliations:** a Department of Neuroscience, Erasmus University Medical Center, the Netherlands; b Department of Psychiatry, Erasmus University Medical Center, the Netherlands; c Department of Applied Ergonomics and Design, Faculty of Industrial Design Engineering, Delft University of Technology, the Netherlands; d Department of Surgery, Erasmus University Medical Center, the Netherlands

**Keywords:** Leap motion controller, Instrument tracking, Surgical skill learning, Surgical education, Laparoscopic training, Haptic feedback, Medical simulation, Cost-effective surgical training

## Abstract

**Background:**

Minimally invasive surgery (MIS) requires mastery of complex skills, for which diverse training methods have been developed. While some methods focus on precise instrument tracking and others on realistic practice scenarios, combining these aspects leads to increased costs and impractical setups.

The Leap Motion Controller (LMC) is a cost-effective device offering precise motion tracking, but previous studies found its utility in surgical training is limited by line-of-sight issues. This study aims to address this challenge.

**Methods:**

A novel interface was developed for use of LMC for tracking MIS instruments during practice. To resolve the line-of-sight problem, the traditional enclosed working area was replaced with a single vertical barrier concealing the task while allowing the LMC to maintain a clear horizontal view of the instrument. Performance metrics included time to task completion and total path length of the instrument. Twenty-eight medical students participated, performing 40 consecutive trials each.

**Results:**

The LMC provided precise tracking, effectively resolving line-of-sight issues. Participants improved significantly, with task completion time decreasing from 61 s (SD = 40) to 19 s (SD = 8) and path length from 2390 mm (SD = 2569) to 574 mm (SD = 348). Performance plateaued after 20 trials, with reduced variance for all outcomes.

**Conclusions:**

The study successfully leveraged the LMC for tracking surgical instruments, overcoming previous limitations. The setup enables real-time monitoring, continuous movement tracking, and tactile interaction with physical objects. Its affordability and simplicity make it a promising tool for traditional and home-based MIS training, especially in resource-limited settings.

## Highlights

• The novel setup overcomes line-of-sight constraints of Leap Motion Controller for minimally invasive surgery training.
• Low cost, simple design supports widespread implementation in clinical programs.
• Promising for both traditional and home-based MIS training in low-resource settings

## Graphical abstract

**Figure:**
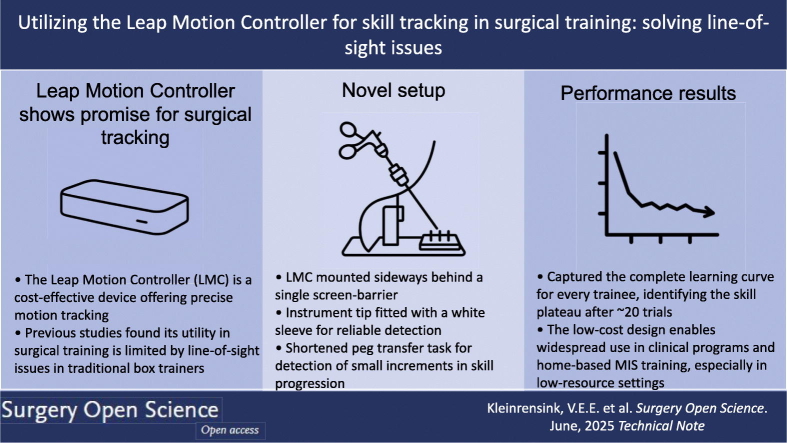

## Introduction

To best prepare surgeons for the demands of the operating room, it is important that they have access to effective tools to enhance their skills outside of it [1]. Particularly in the field of minimally invasive surgery (MIS), the complexity of the procedures requires specialized training methods [2]. In order to ensure that trainees achieve sufficient proficiency in instrument handling prior to clinical application, training methods are required that provide a realistic practice environment and accurate assessment of skill progression. Although various surgical simulators have been developed, there remains a significant lack of access to training opportunities for trainees that can meet both of these requirements, particularly in low-income countries [3,4].

The most accessible training tools, such as laparoscopic box models, primarily track time spent on manual tasks. However, they provide an inadequate representation of instrument control proficiency, as completion time alone may not be sufficient to assess competency [5]. Virtual reality trainers represent a more sophisticated option for tracking skill progress, but they lack tangible object interaction and realistic haptic feedback, which have been shown to be important in minimally invasive surgery [6,7].

To provide both realism and tracking accuracy, several methods have been explored to track instrument motion in physical simulation models, which can be categorized into passive and active systems [8]. Passive systems, such as electromagnetic sensors [9] and ultrasonic transmitters [10], have the advantage that no cables need to be attached to the instrument. These systems meet the requirements of realism and accuracy for training, but their complexity and cost make them less feasible. Therefore, the active variant is the most commonly used solution [8]. A gimbal mechanism is usually used to determine the position of the instrument. These solutions are more user friendly and affordable, but often compromise accuracy due to calibration requirements.

In this respect the optimal application for instrument tracking would be a passive system that is cost effective, easy to implement and accurate. One such system is the Leap motion controller (LMC). LMC is a low cost motion sensor that can track the motion of hands and linear tools in three-dimensional space with an accuracy below 0.2 mm [11]. Several studies have investigated the utility of LMC for tracking in a simulated surgical environment. Lahanas et al. [12] used LMC to track mock handles, which controlled instruments for minimally invasive surgery in a virtual reality program. A limitation of the study was that the measurement of skill progress was limited to orientation in space with the instruments, as the simulated instruments did not have graspers. Partridge et al. [13] used LMC to track the movements of real instruments in a physical box trainer. An advantage of the setup used in this study is that participants were also able to practice physical object manipulation. As a shortcoming of the study, the authors described that it was not possible to follow the instruments directly in the box trainer because LMC requires an unobstructed line of sight to function properly. Therefore, the hands were followed by the sensor instead, resulting in significantly reduced measurement quality. Despite these limitations, both studies demonstrated construct validity, as the LMC was able to accurately discriminate between novices and experts in eye-hand coordination.

Summarizing, there is a clear need for cost-effective and accurate methods to objectively measure performance in minimally invasive surgical training. LMC has emerged as a promising tool in this regard. However, to date it hasn't been widely adopted in surgical training programs, primarily due to a line-of-sight issue that limits its utility. The present study aims to address this issue, with the intention of fully realizing the LMC's ability to improve the accessibility and quality of surgical training. To this end, we will evaluate the effectiveness of a novel setup that allows LMC to directly track the motion of laparoscopic instruments during practice on a physical task.

## Methods

### Apparatus

The setup consisted of an interface for holding the instruments, a computer with a monitor, LMC (Leap Motion Inc., LM-010), a camera, and a software program for capturing and analyzing the data. LMC is a compact sensor that can be connected to the computer via USB 3.0. The dimensions are 13 mm × 13 mm × 76 mm and the weight is 45 g. The device uses three IR LEDS and two CCD cameras. The data is sent out at a rate of up to 120 Hz. The effective range is between 2.5 and 60 cm above the device on the Y axis, and up to about 20 cm in the X- and Z-axes. The accuracy of the sensor was determined to be below 0.2 millimeters mm for static setups [11].

As LMC is not able to recognise and track surgical instruments directly, a white rubber tube was attached to the instrument near the tip, which could be recognised as a pen-shaped object by the sensor. To keep this rubber tube in constant view of LMC while the instrument moved, the sensor was placed on its side, horizontally facing the instrument.

**Fig. 1:**
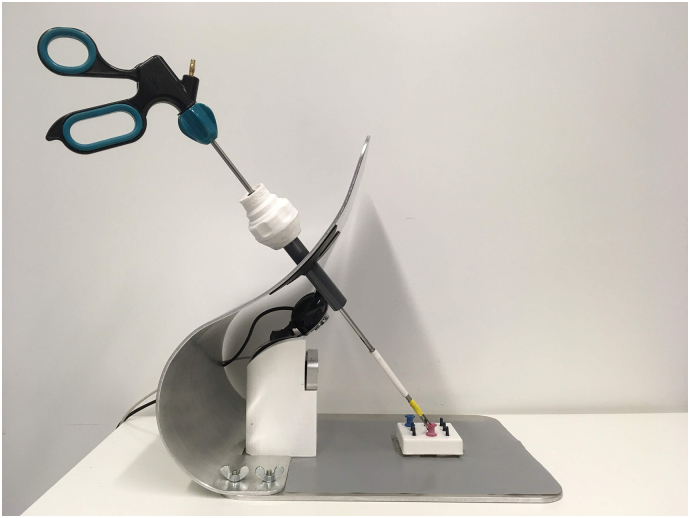
Mechanical interface used in the experiment.

To imitate the conditions of minimally invasive surgeries, it is required that the task is hidden from direct vision. In the traditional box trainer, this is achieved by placing the task in a closed box. However, LMC needs an unobstructed view into the surrounding area to be able to track the objects properly, which means that an enclosed space could not be used. To be able to use LMC, a setup had to be made that provided the sensor a clear line of sight, while simultaneously concealing the working area. This was achieved by placing the work field behind a vertical barrier, which also served as an interface into which mock up trocars could be inserted. Directly in front of this surface a custom made mount was placed, supporting both the sensor and the camera. A webcam (Gemini Gembird) functioned as an endoscope, which was connected to the computer via usb 2.0. The frame rate of the camera was 60 Hz. Version 2.3.1 of the LMC driver software was used to track the motion data. A custom software program, created by OCRAM technologies, implemented in Python version 2.7, was used to extrapolate the motion data from the position of the white tube to the tip of the instrument. The mechanical interface is illustrated in Fig. 1.

A shortened version of the peg transfer task was designed to assess performance. The peg transfer task is part of the Fundamentals of Laparoscopic Surgery program, a validated training course that is a mandatory part of surgical residents training in the United States [14,15]. In this task, 6 pegs are moved to the opposite side of the peg board with grasper forceps, transferring the peg between instruments. In the shortened version designed for this study, participants were asked to move two coloured pegs across the task board, using only the dominant hand. Additionally, the pegs were designed to have six flat surfaces, making these easy to grab.

**Fig. 2:**
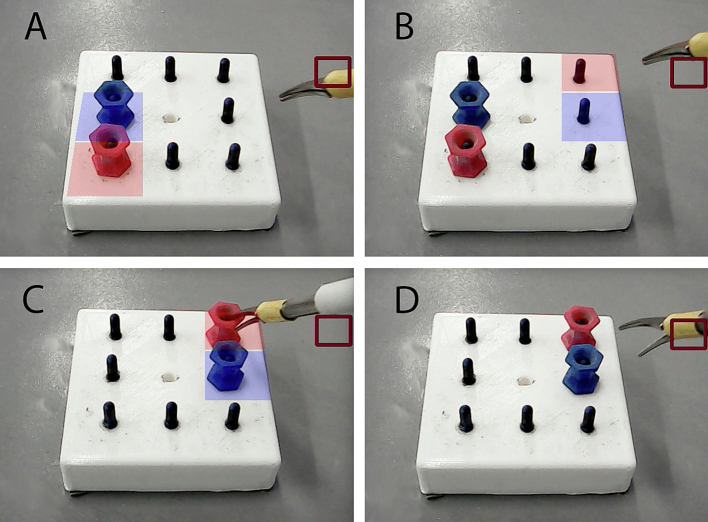
Step-by-step visual sequence of the peg transfer task: A) Initiation of a trial, B) Display of the target configuration, C) Placement of pegs in the designated positions on the task board, D) Completion of the trial and transition to the next one.

Making use of OpenCv 2, the software was able to superimpose figures on the image obtained from the camera, as well as track the position of coloured objects [16]. A sequence of target positions of the pegs was shown on the screen with coloured squares. The sequence was designed to have both pegs travel an approximately equal distance each trial. For left-handed participants, the sequence was presented in mirror image. Each trial began when the instrument tip was moved into a designated start zone on the screen, which was shown as a square in the top right corner for right-handed participants or in the top left for left-handed participants. Entering this area triggered the presentation of the target peg configuration. The trial ended when both pegs were placed in their correct target positions and the instrument was returned to the start zone. This signaled the end of the trial and automatically started the next one. This process is illustrated in Fig. 2.

### Measures

To assess performance, the following parameters were recorded: time to task completion, defined as the total time used to place the two pegs in the correct positions and moving the instrument back to the starting position; complete the peg transfer (define start and end, for example start was first contact with the peg, and end is defined as finalizing contact with the peg); and path length, defined as the total distance traveled by the tip of the instrument during the peg transfer task.

### Participants

Twenty-eight medical students volunteered to participate in the study (18 females, 10 males). The mean age was 18.7 years (SD = 1.2). Twenty-six participants were right-handed, and two were left-handed. All participants had normal or corrected-to-normal vision with no other physical impairments.

### Procedure

Prior to the experiment all participants provided written informed consent. After a demonstration of the task by the experimenter, participants performed 40 consecutive repetitions. This number of repetitions was chosen based on the expectation that participants would be able to complete a learning curve [17] within this range. Participants were instructed to pick up dropped pegs by hand and to continue after placing them in the middle of the task board.

### Statistical analysis

Mean path length, time to completion and standard deviation were computed per repetition. All analyses were carried out with SPSS 21 software.

## Results

**Fig. 3:**
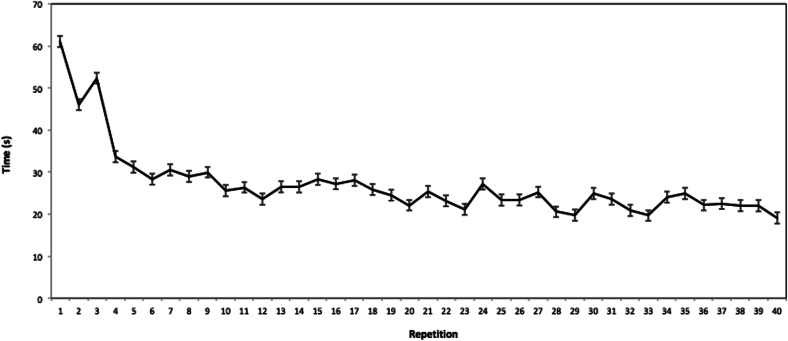
Mean time (in seconds) required to complete each repetition of the peg transfer task. Error bars represent the standard error of the mean.

**Fig. 4:**
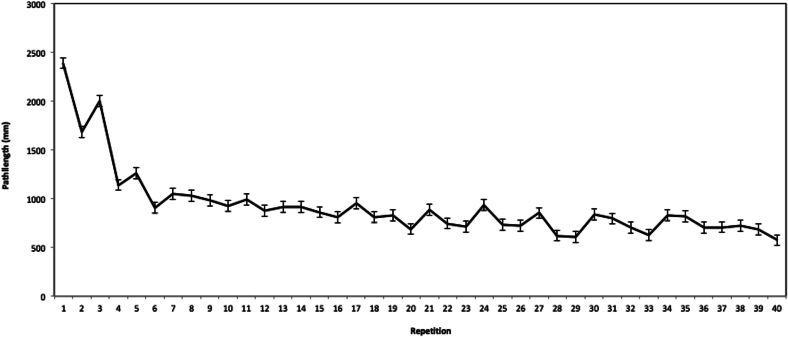
Average path length (in millimeters) traversed by the grasper forceps during each repetition of the peg transfer task. Error bars represent the standard error of the mean.

LMC, applied in the setting as described above, provided precise metrics for instrument control, implicating that line of sight problems were adequately addressed. Mean duration of the total practice session of all participants was 1091 s (18 min and 11 s). At baseline, participants required 61 s on average (SD = 40 s) to complete the task and had an average path length of 2390 mm (SD = 2569 mm). At the last trial, participants required 19 s on average (SD = 8 s) to complete the task and had an average path length of 574 mm (SD = 348 mm). A repeated measures ANOVA showed a significant reduction in path length across trials, F(39, 1053) = 4.34, p < .001, indicating improved instrument control. No significant change was found in completion time, F(9, 243) = 0.85, p = .575. On average, performance approached the lower asymptotic level on both time to completion and total path length after 20 trials. The mean changes of all participants in task completion time and total path length with increasing experience are shown in Fig. 3, Fig. 4 respectively.

## Discussion

The aim of this study was to evaluate the efficacy of a novel setup that allows the LMC to track the progression of basic laparoscopic skills in a physical task. The setup allowed direct monitoring of instrument positions, allowing continuous tracking of movements while providing haptic feedback to the trainee during practice. The system accurately mapped small increments in the learning curve up to the plateau phase for all participants, indicating with confidence when a participant had achieved the necessary skill level for the task.

An added benefit of this direct tracking feature is the ability to initiate and stop task tracking by moving an instrument to a specific location. This eliminates the need for manual intervention during practice sessions, allowing for more accurate measurement of progress on a trial-by-trial basis. This feature contributes significantly to the ability to determine when students reach a plateau in their learning. Such insights allow for a more personalized approach to training, advancing students to increasingly complex tasks that match their current skill level. This may also improve motivation by preventing stagnation and keeping trainees challenged. Furthermore, because of the low cost of this setup, it may become possible for multiple students to have individual practice stations during a training session, enabling students to practice at their own pace.

The potential applications of the LMC setup extend to a variety of training environments. The simplicity of the setup, requiring only a barrier to obscure the direct view of the task and a holder for the sensor, makes it easy to fabricate, disassemble, and transport. Its portability allows for convenient use at home, extending training opportunities beyond traditional environments.

### Limitations

While the results of this study are promising, several limitations must be acknowledged. First, to allow efficient testing of the tracking system and observe skill progression up to a performance plateau within a short timeframe, we used a simplified, one-handed version of the peg transfer task. Future research should examine whether the findings generalize to more complex tasks, such as cutting or bimanual instrument use.

Second, we used a standard webcam instead of a surgical camera to avoid introducing multiple challenges at once. This allowed us to focus solely on the progression of hand–eye coordination and managing the fulcrum effect. Real laparoscopic imaging is more challenging due to a smaller, moving field of view. Future studies could examine how to assess skill in this area once basic instrument control has been mastered.

Third, the present study utilized the Leap Motion Controller V2. Although a newer version of the LMC has been introduced to the market, both versions rely on infrared cameras for tracking. Consequently, regardless of which model is used, a clear line of sight is required for effective tracking. This limitation is inherent to the technology, meaning that the latest version also requires a setup that addresses the challenges described in this study.

## Conclusion

This study demonstrated that the novel setup incorporating the Leap Motion Controller (LMC) effectively overcomes the line-of-sight limitations that previously hindered its use in minimally invasive surgery (MIS) training. The low cost and simplicity of the setup make it a promising tool for widespread use in both traditional and home-based training environments, particularly in resource-limited settings.

## CRediT authorship contribution statement

**V.E.E. Kleinrensink:** Writing – original draft, Visualization, Software, Project administration, Methodology, Investigation, Formal analysis, Data curation, Conceptualization. **R.H.M. Goossens:** Writing – review & editing, Supervision, Methodology, Conceptualization. **J.F. Lange:** Writing – review & editing, Supervision, Conceptualization. **L.W. Kranenburg:** Writing – review & editing, Supervision, Conceptualization. **G.J. Kleinrensink:** Writing – review & editing, Supervision, Resources, Methodology, Conceptualization.

## Ethical approval

Ethical approval for the study was granted by the medical ethical committee of Erasmus University Medical Center Rotterdam.

## Funding

This research did not receive any specific grant from funding agencies in the public, commercial, or not-for-profit sectors.

## Declaration of competing interest

The authors have no relevant financial or non-financial interests to disclose.
